# A third hand to the surgeon: the use of an endoscope holding arm in endonasal sinus surgery and well beyond

**DOI:** 10.1007/s00405-021-06935-x

**Published:** 2021-06-20

**Authors:** Constantin A. Hintschich, René Fischer, Caroline Seebauer, Karl-Michael Schebesch, Christopher Bohr, Thomas Kühnel

**Affiliations:** 1 Department of Otorhinolaryngology, University of Regensburg, Franz-Josef-Strauß-Allee 11, 93053 Regensburg, Germany; 2 Department of Neurosurgery, University of Regensburg, Regensburg, Germany

**Keywords:** Paranasal sinuses, Skull base, Endoscopic surgery, Hypopharynx, Oropharynx, Larynx, Transoral approach

## Abstract

**Background:**

Extended endoscopic endonasal operations of the sinuses and the frontal skull base require a bimanual action of the surgeon in many cases. Thus, typically an assistant guides the endoscope and centers the field of view. In this study, we investigate in which cases an endoscope holding arm can be used alternatively.

**Materials and methods:**

The electromagnetic system ENDOFIXexo was used in different surgical interventions of the paranasal sinuses and beyond questioning ergonomics and geometrical limitations. The realized degrees of freedom were documented, and a topography of possible applications compiled.

**Results:**

The presented system is limited by the anatomy of the anterior ethmoid and dynamic working conditions in the sagittal direction. Especially in extended interventions in the posterior ethmoid, in which parts of the nasal septum have been resected and a static position of the endoscope is desired the surgeon can greatly benefit from the robotic arm. Moreover, through the high flexibility of the endoscopic arm surgeries of the pharynx and larynx were performed, questioning the current gold standard of microscope-assisted surgical procedures.

**Conclusion:**

Under the impression of an urging staff shortage and due to its unlimited patience, the ENDOFIXexo arm seems promising. Taking into account the complex anatomy and the limited access, we especially see a favorable field of application in the surgery of the pituitary gland and skull base tumors.

## Introduction

Enhanced instruments, improved visualization through HD cameras and monitors and intraoperative stereotactic navigation systems pushed the boundaries much further [14]. By now, most interventions of the paranasal sinuses and an increasing part of frontal skull base surgery can be managed endoscopically [10]. Even pathologies beyond the skull base such as pituitary gland tumors [8], clivus chordomas [16] and basilar apex aneurysm [9] can be treated by an endoscopic endonasal approach.

One major disadvantage of endoscopic surgery is that the surgeon can only operate with one hand whilst holding the endoscope with the second hand. However, in extended interventions or critical situation, bimanual action is often highly desired or even necessary [2]. Commonly, this can be achieved by a four hands technique in which the assistant guides the endoscope. However, the integration of the co-surgeon and a third and fourth hand has generally a flat learning curve [3]. The static activity of holding the endoscope during long-lasting interventions leads often to a tremor of the assistant, consequently to a shaky image and eventually smudging of the endoscope lens with blood [13]. Additionally, in times of a staff shortage and the economization of health care there is not always an assistant available.

However, there have been various approaches to ease bimanual action by endoscope holding arms during the last decades [1, 11], Friedrich et al. 2019). The basic design consists of a multi-membered arm with either mechanical, pneumatic or electromagnetic locks. Although most of these endoscope holders include no active motion, the recent AESOP system (Medineering, Munich, Germany) is automated and controlled by the surgeon through a foot pedal in a hands free manner (Friedrich et al. 2019).

Nevertheless, several disadvantages have been revealed in existing products [1, 11]: downwards drift of the arm, high costs of more elaborated holders, iatrogenic trauma through the fixed endonasal endoscope in the patient’s head, bulky design of the arm and inflexibility through limited degrees of freedom compared to free hand movement. This might be why, by now no of the current endoscope holding devices could prevail neither in otolaryngology nor in neurosurgery.

We report here on the new endoscope holding arm ENDOFIXexo (AKTORmed, Barbing, Germany) which was originally designed for abdominal procedures and successfully modified for sinus surgery [7].

## Materials and methods

Between May 2019 and December 2020, 30 endoscopic surgeries were performed using the ENDOFIXexo in the ENT department of the university hospital of Regensburg, Germany (see Table 1). There was no single complication caused by the use of the ENDOFIXexo system. The presented cases were partly collected retrospectively and partly documented prospectively.

**Table 1:** Surgeries performed using the ENDOFIXexo system

Case	Age	Sex	Diagnosis	Surgery	Approach	Mononostril/binostril
1	55	M	Sinunasal polyposis	FESS, DRAF III	Endonasal	Binostril
2	43	M	Recurrent sinunasal polyposis	FESS, DRAF III	Endonasal	Binostril
3	47	F	Pituitary adenoma	Pituitary adenomectomy	Transsphenoidal	Binostril
4	47	F	Meningocele with rhinoliquorrhea	Meningocele, DRAF III, Frontobasal duraplasty	Endonasal	Binostril
5	49	M	Adenocarcinoma of the parasnasal sinuses with Optic nerve compression	Tumor resection, decompression of the optic nerve, frontobasal duraplasty	Endonasal	Binostril
6	68	M	Pituitary adenoma	Pituitary adenomectomy	Transsphenoidal	Binostril
7^a^	14	M	Juvenile nasopharyngeal fibroma	Resection after previous embolisation	Prelacrimal-transmaxillar, Transsphenoidal	Mononostril
8	53	F	Meningocele with rhinoliquorrhea	Meningocele resection, frontobasal duraplasty	Endonasal	Binostril
9	75	M	Pituitary adenoma	Pituitary adenomectomy	Transsphenoidal	Binostril
10	67	F	adenocarcinoma of the Sphenoid sinus and clivus	Tumor resection	Transsphenoidal	Binostril
11	45	F	Pituitary adenoma	Pituitary adenomectomy	Transsphenoidal	Binostril
12	61	M	Pituitary adenoma	Pituitary adenomectomy	Transsphenoidal	Binostril
13	50	F	Pituitary adenoma	Pituitary adenomectomy	Transsphenoidal	Binostril
14	31	M	Pituitary adenoma	Pituitary adenomectomy	Transsphenoidal	Binostril
15	57	M	Recurrent adenocarcinoma of the Parasnasal sinuses	Tumor resection, Frontobasal duraplasty	Endonasal	Binostril
16	20	M	Juvenile nasopharyngeal fibroma	Resection after previous embolisation	Endonasal	Mononostril
17	52	M	Recurrent sinunasal polyposis	FESS	Endonasal	Mononostril
18	61	M	Pituitary adenoma	Pituitary adenomectomy	Transsphenoidal	Binostril
19^a^	79	F	Esthesioneuroblastoma	Tumor resection, DRAF III, frontobasal duraplasty	Endonasal	Binostril
20	60	F	Mucocele of the left frontal sinus	Resection of the mucocele, DRAF III	Combined endonasal and external	Binostril
21	53	F	Meningocele with rhinoliquorrhea	meningocele resection, Frontobasal duraplasty	Endonasal	Binostril
22^a^	60	M	Pituitary adenoma	Pituitary adenomectomy	Transsphenoidal	Binostril
23^a^	60	M	Parapharyngeal metastasis of an esthesioneuroblastoma	Resection of the metastasis	Transoral	–
24	61	M	Pituitary adenoma	Pituitary adenomectomy	Transsphenoidal	Binostril
25	54	M	Recurrent adenoid cystic carcinoma of the nasopharynx	Tumor resection	Transnasal, Transoral	–
26	79	M	Postoperative synechia of the anterior commissure after T1 larynx carcinoma	Dissection and treatment with Mitomycin C	Transoral	–
27^a^	68	F	Squamous cell carcinoma of the dorsal hypopharynx wall	Tumor resection	Transoral	–
28	23	F	Schwannoma of the olfactory fibres with intracranial extention	Tumor resection	Endonasal	Binostril
29	71	M	Pituitary adenoma	Pituitary adenomectomy	Transsphenoidal	Binostril
30	72	M	Squamous cell carcinoma of the right vocal cord	Tumor resection	Transoral	–

^a^Cases enlightend in more detail in the results section

### Robotic endoscopic arm system

**Fig. 1:**
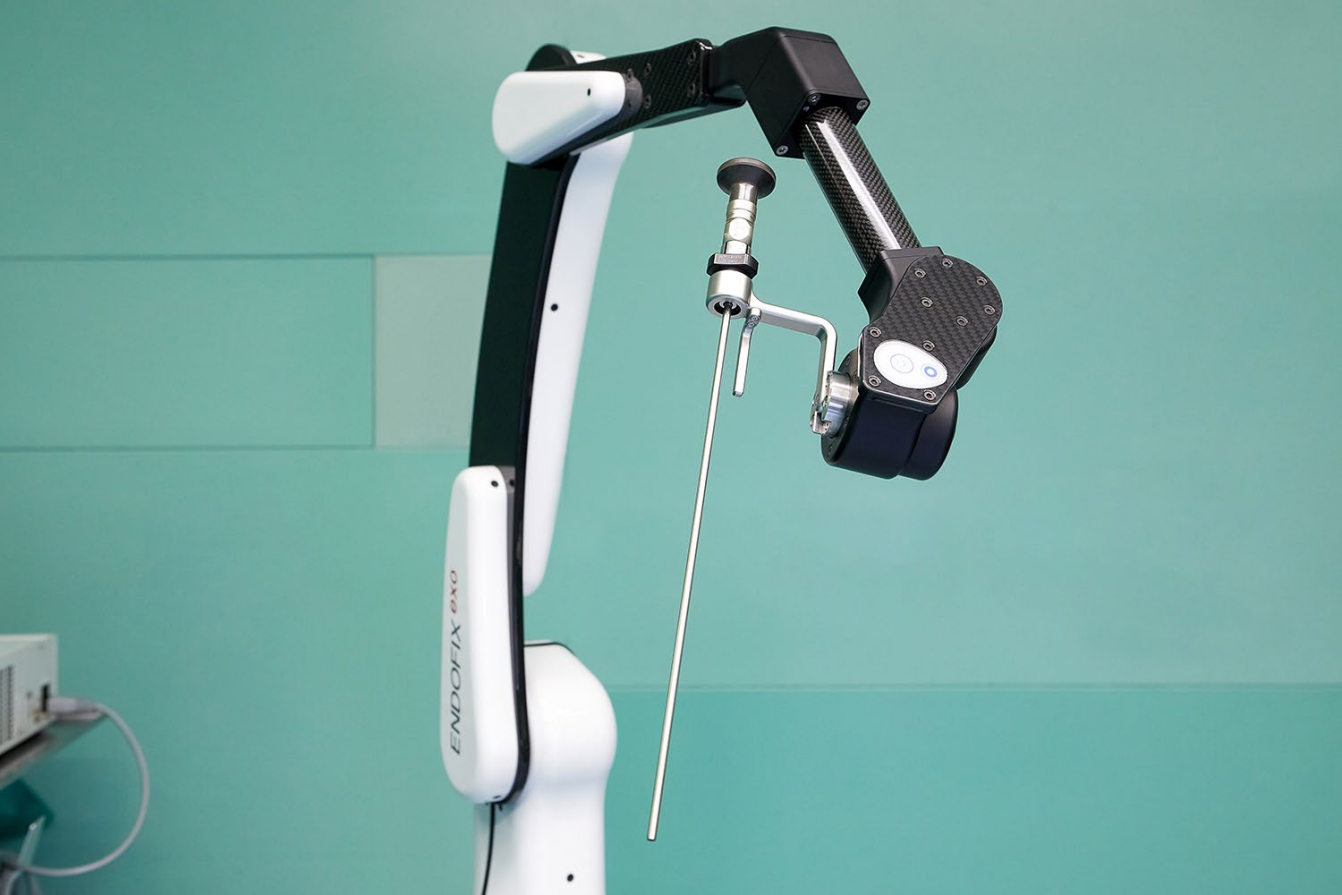
The three electromagnetic locks of the ENDOFIXexo allow any position within arm range

The ENDOFIXexo is an electromagnetic manual support arm to hold the endoscope (Fig. 1). It consists of three segments and three step-less electromagnetic locks allowing six degrees of freedom. Thus, any position within the range of the arm can be reached. When pushing the control button on the distal part of the arm all electromagnetic locks open and the system can easily be maneuvered as in standard endoscopic endonasal surgery. As soon as the control button is released, the locks immediately fix again. The maximum working load is 1 kg.

The system can easily be attached to the standardized side rails of an operating table. Sterile conditions are assured through a sterile single-use plastic cover and an autoclavable endoscope clamp. This endoscope clamp can hold any standard rigid endoscopes.

### Surgical setup

**Fig. 2:**
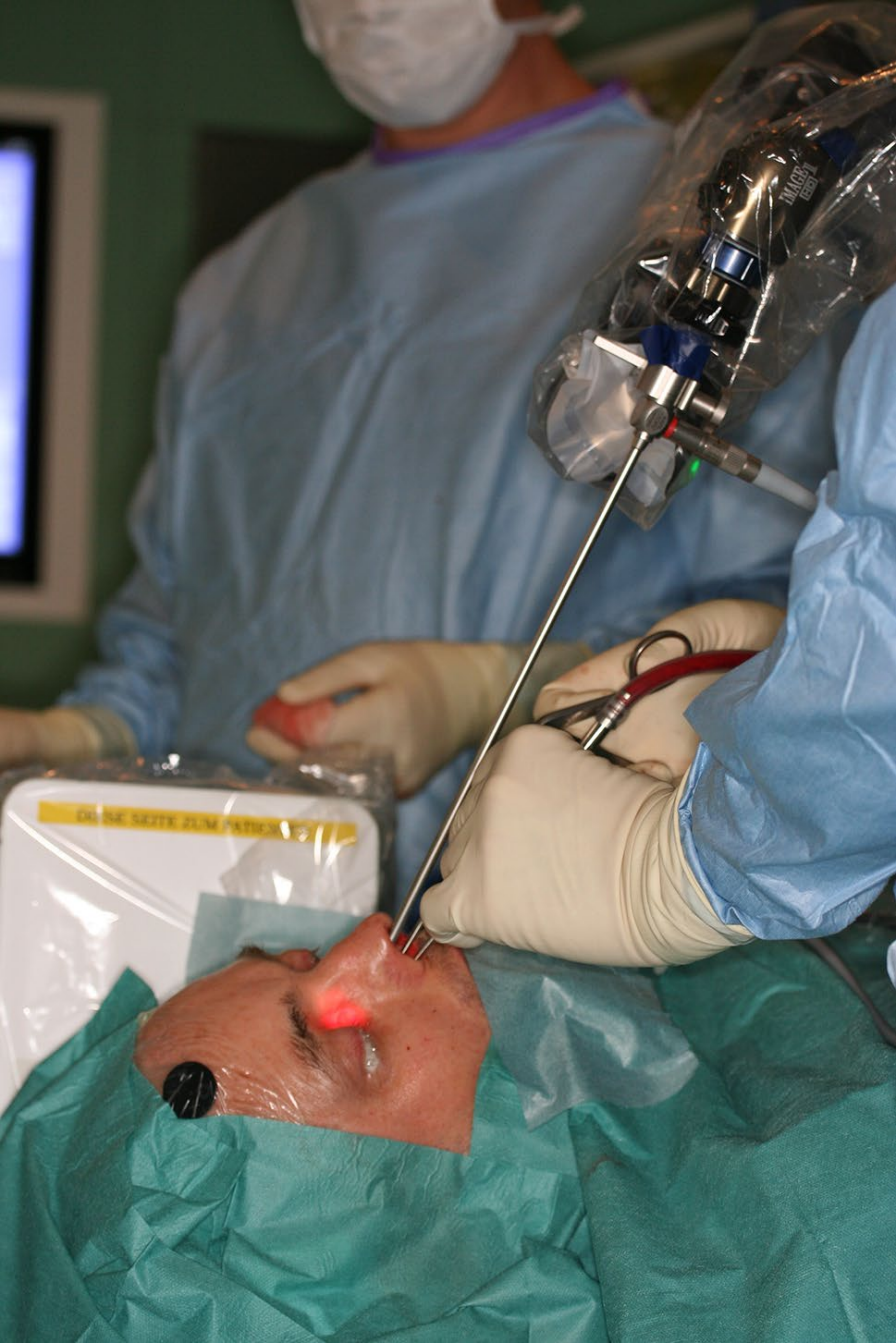
ENDOFIXexo arm with endoscope of 30 cm length eases bimanual action

The patient was placed on the operating table in a supine position with the head slightly elevated. The ENDOFIXexo system was fixed to the rails on the left side of the operating table. After skin disinfection and surgical draping of the patient the endoscopic arm was covered with the single-use cover. Then, thes autoclaved endoscope clamp was mounted, and the endoscope attached to it. As compared to a Hopkins endoscope of a length of 19 cm in free hand endoscopic endonasal surgery a 30 cm endoscope had to be chosen to allow an unrestricted movement of the surgeon’s hands (see Fig. 2). However, the longer 30 cm Hopkins endoscope has a wider diameter of 5 mm compared to the endoscope with a diameter of 4 mm.

## Results

### Cases

All 30 surgeries performed using the ENDOFIXexo are listed in Table 1. Following, we present five illustrative cases in more detail:

#### Case 7—Juvenile nasopharyngeal fibroma

**Fig. 3:**
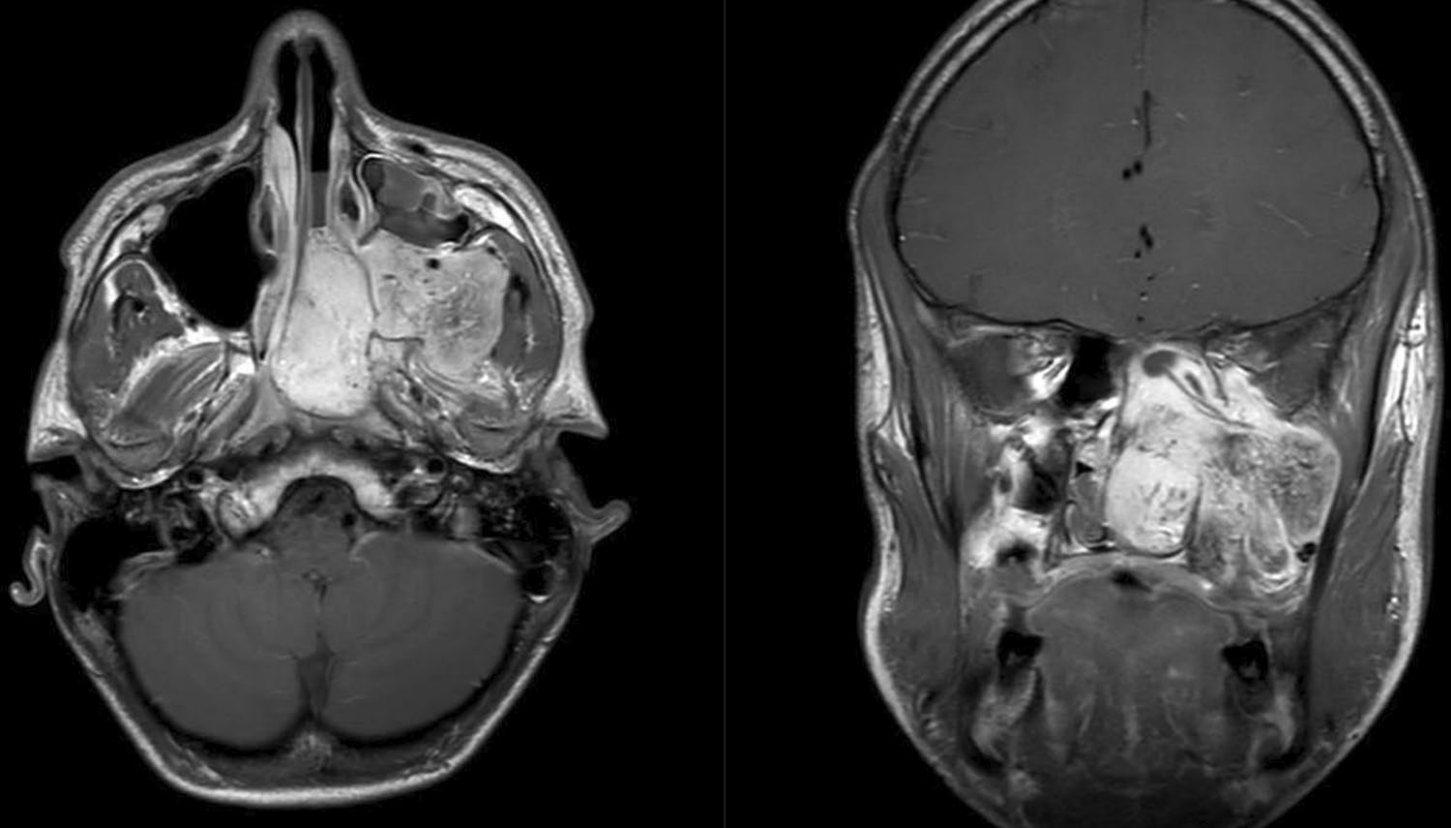
MRI of the juvenile nasopharyngeal fibroma in the left nasal cavity, nasopharynx and pterygopalatine fossa

A boy of 14 years presented with a nasal obstruction and left side rhinorrhoea. A MRI showed a massive destructing process in the left nasal cavity, nasopharynx and pterygopalatine fossa, most compatible with a juvenile nasopharyngeal fibroma (Fig. 3).

The day before surgery, the afferent vessels were embolized to minimize intraoperative bleeding. A prelacrimal approach was chosen to access the tumor. After the opening of the posterior wall of the maxillary sinus, the ENDOFIXexo system was installed to allow the surgeon to operate in a bimanual action. This was mandatory to conduct a sufficient hemostasis of the despite embolisation still diffusely bleeding tumor. The tumor was extirpated in the nasal cavity, both the maxillary and sphenoid sinus, the nasopharynx, the pterygopalatine fossa and the cavernous sinus. The maxillary artery was ligated, the infraorbital and the optical nerve as well as the internal carotid artery were identified and preserved.

#### Case 19—Esthesioneuroblastoma

A female patient aged 79 years presented with aqueous rhinorrhea, anosmia and recurrent epistaxis. A CT revealed a substantial process of the ethmoid and nasal cavity. A transnasal biopsy provided the diagnosis of an esthesioneuroblastoma.

The intranasal part of the tumor was taken out in a standard one-handed endoscopic technique. The cranial septum occurred to be infiltrated by the tumor and was therefore resected. Using the resulting binostril approach the endoscope holding arm was installed and the operation continued in a two hands technique: The cranial tumor parts were removed, and the adjacent skull base was resected. The underlying dura, the olfactory bulb and the gyrus rectus were infiltrated by the tumor and therefore removed. Frozen sections ensured tumor free margins. The resulting skull base defect was reconstructed in an underlay technique using fascia lata, fibin glue and commercial collagen tissue.

#### Case 22—Pituitary adenoma

A 60-year-old patient presented himself with headache, tingling paraesthesia of the right arm and diplopia. An MRI revealed a tumor adjacent to the optic chiasm highly suspicious of an adenoma of the pituitary gland.

The resection through a binostril transnasal endoscopic approach was conducted as described elsewhere [12]: Following the bilateral opening of the sphenoid sinus through the natural ostium, the intersphenoid septum and the dorsal nasal septum were resected. After the creation of a sufficiently large access to the sphenoid sinus, the robotic arm was placed, and the endoscope focused. In a bimanual action the dorsal mucosa of the sphenoid sinus was incised, the underlying bone of the sella floor partly removed and the capsule was opened to access the adenoma. Then the adenoma was removed in a suction/curettage-technique. The immunohistochemical analysis confirmed a pituitary adenoma.

#### Case 23—Parapharyngeal metastasis of an esthesioneuroblastoma

**Fig. 4:**
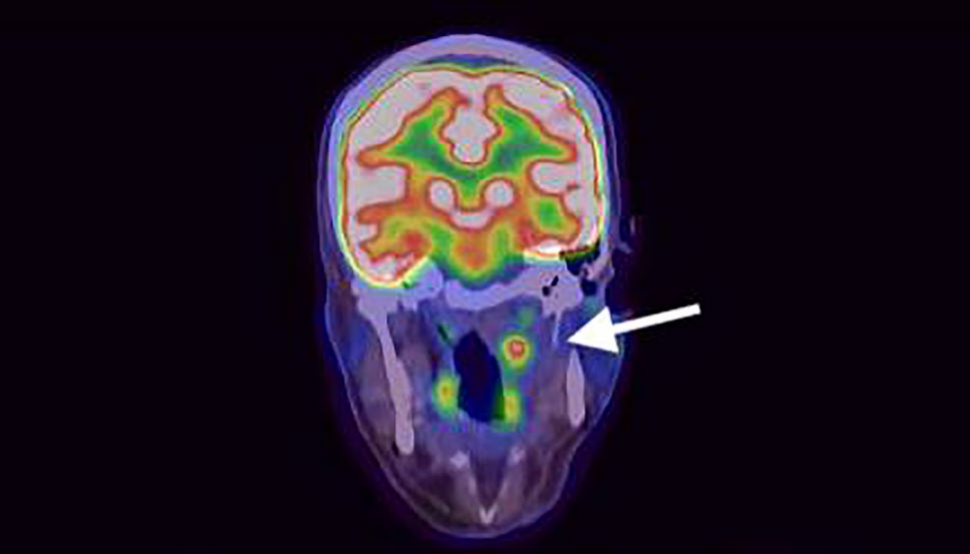
PET/CT with FDG positive mass of the left cranial parapharyngeal space

A male patient of 43 years with a long history of an esthesioneuroblastoma and repeated episodes of tumor recurrence presented with a mass of the left cranial parapharyngeal space, highly suspicious for a lymph node metastasis. Although previously stereotactically irradiated a subsequent PET/CT revealed still FDG uptake (see Fig. 4). A salvage surgery was strongly indicated despite the critical anatomical location in the nasopharynx, just ventrally of the vertebral column and adjacent to the large cervical vessels.

As generally known, it is challenging to approach the nasopharynx for surgery. Therefore, we chose a transoral access using an endoscopic technique and the ENDOFIXexo system to ensure a bimanual procedure. After the setup of the robotic arm guiding, a 30° endoscope the mucosa was incised and the tumor was carefully dissected out of the surrounding tissue. Despite scar tissue and the close proximity to the internal carotid artery, the node could be removed entirely. The result of the histopathological examination confirmed the suspected diagnosis. Here, as in most open surgery of the head and neck and hence in contrast to standard FESS procedures, a bimanual action was required.

#### Case 27—Squamous cell carcinoma of the dorsal hypopharynx wall

A woman of 68 years was referred to our hospital with a histopathologically confirmed squamous cell carcinoma of the right dorsal hypopharynx wall. After exclusion of a second carcinoma through triple endoscopy and an unsuspicious staging with chest CT and abdominal sonography, the tumor resection and a bilateral neck dissection were planned.

**Fig. 5:**
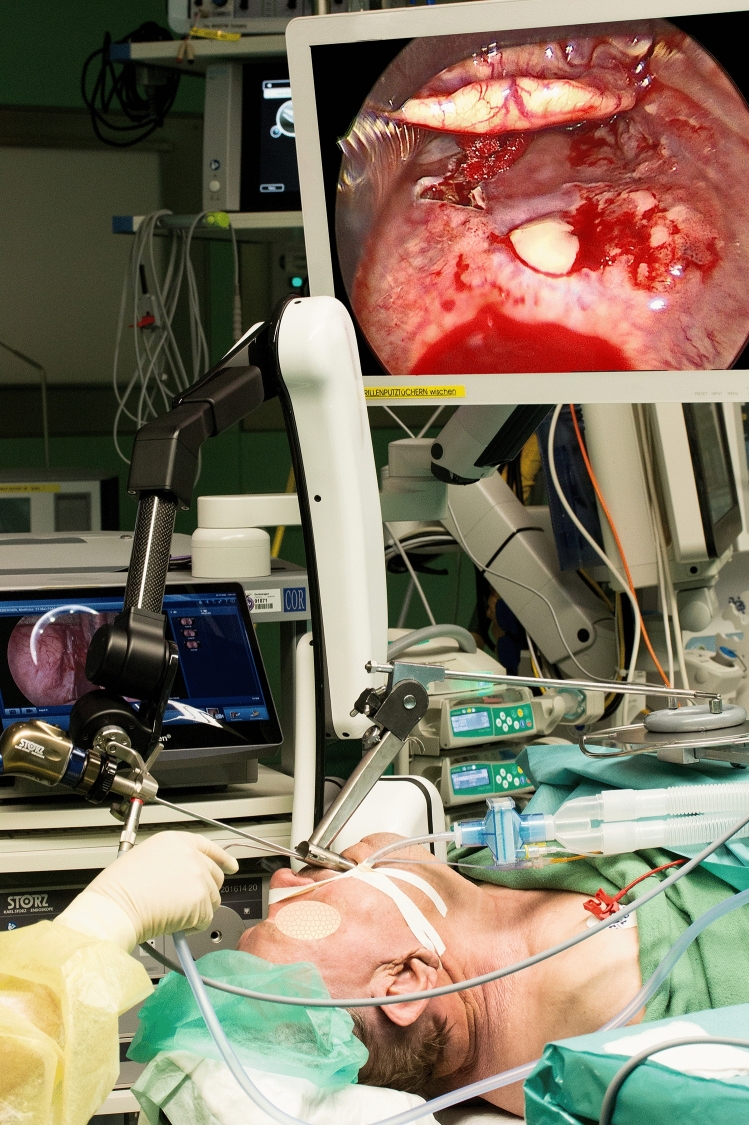
Transoral endoscopic resection of the squamous cell carcinoma of the hypopharynx

To test the limits of the ENDOFIXexo system and to question the current gold standard of microscopic laser surgery, we chose an endoscopic transoral approach: after general anesthesia and intubation, a laryngoscope was inserted transorally to expose the entire tumor of 2 × 3 cm size. A 0° endoscope, which was attached to the holding arm, was positioned and allowed a very detailed visualization (see Fig. 5). Monopolar diathermy was employed to resect the tumor down to the prevertebral fascia. Frozen section revealed carcinoma in situ at the caudal mucosal margin. Here additional tissue was removed, and a careful hemostasis carried out. Subsequently, the bilateral neck dissection was performed in a standard open approach.

### Anatomical considerations for endonasal application

As described above, a Hopkins endoscope of 30 cm length and 5 mm diameter (Karl Storz, Tuttlingen, Germany) was used to allow maximal bimanual handling to the surgeon (see Fig. 2). However, the narrow anatomy of the anterior ethmoid limits the use 5 mm endoscope attached to the endoscope holding: The anterior ethmoid is 7 mm wide only and, therefore, hardly accessible [6]. Caudally, the funnel of the orbit taper and the ethmoid opens laterally up to 15 mm width. Thus, in a mononostril approach, only the cranio-posterior ethmoid and the sphenoid sinus are well accessible—especially in a bimanual action. If the nasal septum is resected additionally—as in extended skull base tumors with tumor infiltration of the septum or modified Lothrop procedures—the binostril approach enables the surgery of the entire cranial ethmoid and the sphenoid as well as to some extent the frontal sinus.

## Discussion

The ENDOFIXexo system is a passive endoscope holding arm, allowing bimanual endoscopic action in various sites of ENT surgery. It combines three fundamental requirements to an endoscope holding arm: intuitive maneuverability, flexibility, and high stability [11].

In comparison to some competitors, the ENDOFIXexo does not have any controlled active motion. Through the push and hold button, the arm becomes flexible and can manually be moved including the attached endoscope. This passivity allows for a straightforward handling and decreases the risk of accidental skull base perforations caused by controlling errors. The electromagnetic locks have indefinite fixing options within its range. Together with the six degrees of freedom any endoscope position within the accessibility can be reached. A high level of stability is guaranteed through the described electromagnetic locks and supports up to 1 kg, fully sufficient to hold the endoscope and the camera.

The ENDOFIXexo system enables perfectly a bimanual action in endoscopic interventions. This eases critical situations in transnasal surgery of the paranasal sinuses and adjacent structures as seen in our cases. Moreover, supporting the surgeon to operate with both hands the ENDOFIXexo arm also extends the possible applications of endoscopic surgery in ENT itself. As highlighted in our examples, we performed endoscopic surgery of the pharynx and larynx. In these cases, the endoscope holding arm showed a high extent of motion and allowed a perfect endoscopic visualization of the situs.

Compared to many other innovations in medical technology, this endoscope holding system is economical through low running costs: the fixing clamp can be sterilized and besides a simple plastic cover no other single-use products are necessary for the utilization. On the contrary, total operating time and hence, costs can be reduced through time-saving bimanual surgery and the rapid set up of the holding arm [2].

However, the ENDOFIXexo system has some important limitations besides the restricted accessibility of the anterior ethmoid (see results): The dynamic preparation in sagittal direction require an ongoing change of the endoscope position. Hence, the system has to be readjusted frequently, which might prolong the duration of surgery compared to a free hand movement. Skull base perforation could be caused by a slight movement of the patients head relative to the fixed endoscope. This is why it is essential, that a deep anesthesia is maintained throughout the entire operation. Accordingly, this has to be mentioned in the preoperative team time out.

As the endoscope is fixed over a longer time compared to standard FESS its shaft might cause pressure lesions of the highly vulnerable endonasal mucosa or the vestibular epithelium. This can be prevented through a cautious orientation of the endoscope and potentially a frequent repositioning.

Despite of the two-dimensional endoscopic picture as compared to three-dimensional microscopic visualization, the surgeon will highly benefit from the better illumination, the wide-angle and the view depth of field. This endoscope holding arm and possibly the upcoming introduction of 3D endoscopy could support the implementation of endoscopic surgery far beyond the paranasal sinuses and the anterior skull base [15]. A similar development took place in transnasal surgery of the pituitary gland: Here endoscopy succeeded over microscopy within the last decades significantly improving the postoperative outcome [5].

## Conclusion

The presented ENDOFIXexo endoscope holding arm unites perfectly intuitive maneuverability, flexibility and stability. Thus, the surgeon can operate in a bimanual action—even in absence of an assistant. However, in endonasal surgery, the accessibility is restricted to the posterior ethmoid and the sphenoid sinus or dependent on the partial resection of the nasal septum. With a bimanual action, endoscopic surgery may not be limited to paranasal sinuses and the frontal scull base, but expand to other operating sites such as the pharynx and larynx.
